# Effect of *ABCA1* promoter methylation on premature coronary artery disease and its relationship with inflammation

**DOI:** 10.1186/s12872-021-01894-x

**Published:** 2021-02-08

**Authors:** Fang An, Chao Liu, Xiujuan Wang, Tan Li, Hao Fu, Buhe Bao, Hongliang Cong, Jihong Zhao

**Affiliations:** 1 Graduate School, Tianjin Medical University, Tianjin, 300070 China; 2 Department of Military General Medicine, Characteristic Medical Center of Chinese People’s Armed Police Force, Tianjin, 300162 China; 3 Institute of Cardiovascular disease, Tianjin Chest Hospital, Tianjin, 300222 China; 4 Institute of Cardiovascular Disease, Characteristic Medical Center of Chinese People’s Armed Police Force, Tianjin, 300162 China; 5 Department of Pathogen Biology, Logistics University of Chinese People’s Armed Police Force, Tianjin, 300309 China; 6 Department of Clinical Laboratory, Characteristic Medical Center of Chinese People’s Armed Police Force, Tianjin, 300162 China; 7 Department of Cardiology, Tianjin Chest Hospital, Tianjin, 300222 China

**Keywords:** DNA methylation, HDL cholesterol, Coronary artery disease, Neutrophil extracellular traps, ATP-binding cassette transporter

## Abstract

**Background:**

ATP-binding cassette transporter A1 (ABCA1) plays a major role in high-density lipoprotein (HDL) metabolism and reverse cholesterol transport (RCT) and exerts anti-inflammatory effects. Increased *ABCA1* promoter methylation level may result in the progression of coronary artery disease. Thus, the present study investigated the association between promoter methylation status of *ABCA1* and inflammation in the development of premature coronary artery disease (pCAD).

**Methods:**

PCAD patients and healthy individuals (*n* = 90 each) were recruited from the Characteristic Medical Center of the Chinese People's Armed Police Force from June to December 2019. Using pyrosequencing, the levels of *ABCA1* promoter methylation in their blood samples were evaluated. Serum concentrations of lipids, interleukin 1β (IL-1β), C-reactive protein (CRP), and circulating free DNA/Neutrophil extracellular traps (cfDNA/NETs) were also routinely measured and compared between the two groups. *P *values < 0.05 were considered statistically significant.

**Results:**

The mean *ABCA1* promoter methylation levels were significantly higher in the pCAD group than in the control group (44.24% ± 3.66 vs. 36.05% ± 2.99, *P* < 0.001). Based on binary logistic regression analysis, *ABCA1* promoter methylation level was identified as an independent risk factor for pCAD development (odds ratio = 2.878, 95% confidence interval: 1.802–4.594, *P* < 0.001). Furthermore, *ABCA1* promoter methylation levels were negatively correlated with HDL levels (*r* =  − 0.488, *P* < 0.001) and positively correlated with the levels of CRP, cfDNA/NETs, and IL-1β (*r* = 0.389, 0.404, 0.385, respectively; *P* < 0.001). Multiple regression analysis showed that the serum levels of CRP, IL-1β, and cfDNA/NETs independently affect *ABCA1* promoter methylation.

**Conclusions:**

Our findings indicate that high methylation levels at the *ABCA1* promoter are associated with low HDL cholesterol levels and an increased risk of pCAD. Inflammatory factors and NETs may be involved in the progression of pCAD by affecting *ABCA1* promoter methylation levels.

## Background

Despite the continuous improvement in preventive measures and medical technology, coronary artery disease (CAD) remains as the main cause of mortality worldwide [1, 2]. Its age of onset is gradually becoming younger, especially in developing countries, and the incidence of premature coronary artery disease (pCAD) is yearly increasing [3, 4]. Because pCAD has a long course and poor prognosis, its prevention and treatment is an urgent medical problem that needs to be addressed. However, the definition of pCAD was not uniformly established, the age cut-off can vary from 45 to 65 years among studies due to the variation in risk factors affecting different populations and ethnicities [3–6]. Chinese Studies on pCAD commonly used the definition of onset age < 55 years for males and < 65 years for females [7].

Various genetic and environmental factors have been identified to contribute to pCAD development [8]; in particular, multiple genes related to lipid metabolism play a major role in the development of CAD [9]. The ATP-binding cassette transporter A1 (ABCA1) is an integral membrane protein belonging to the ATP-binding cassette membrane transporter family, and the human *ABCA1* gene is located on chr9q31, having a total length of 149 kb and including 50 exons and 49 introns [10]. It mediates cholesterol transfer from the periphery to the liver and intestine in a process called reverse cholesterol transport (RCT), which is critical in promoting the synthesis of high-density lipoproteins (HDLs) and maintaining cholesterol homeostasis and delaying the progression of atherosclerosis (AS)[11].

DNA methylation is an epigenetic phenomenon that typically inhibits gene expression and is characterised by the introduction of an activated methyl group onto the fifth carbon atom of cytosine catalysed by DNA methyltransferase (DNMT) [12]. In recent years, growing evidence has revealed that DNA methylation regulates the expression of specific genes, affects blood lipid metabolism, and participates in the development of AS [13]. However, these studies have mainly focused on patients with familial hypercholesterolemia (FH), whereas those with pCAD are less frequently investigated. Moreover, data on the relationship between serum lipid levels and the methylation degree of the *ABCA1* promoter have been inconsistent and conflicting [14, 15].

Inflammatory response is closely related to the occurrence and development of AS [16]. ABCA1 has been recently found to exhibit anti-AS activity by mediating cellular lipid efflux and suppressing inflammatory response via modifying membrane fluidity or directly activating inflammatory pathways and inhibiting the expression of inflammatory factors [17]. Contrarily, a variety of inflammatory factors (such as TNF-α, IL-1β, and INF-γ) and proteins (such as CRP) have been determined to exert complex regulatory effects on the expression of ABCA1 [18].

Neutrophil extracellular traps (NETs) are extracellular fibres composed of DNA, histones, and microbicidal granular proteins and play important roles in antimicrobial responses as they can effectively trap pathogens, thus preventing infections [19]. Although NETs can inhibit inflammatory responses by promoting the degradation of cytokines and chemokines, they can also have proinflammatory effects. Recently, the role of NETs in AS has been revealed, NETs can induce endothelial cells, lead to pro-inflammatory immune responses, and participate in atherosclerotic formation [20, 21]. However, there are only a few studies investigating the influence of inflammatory mediators and NETs on the expression and methylation status of the *ABCA1* promoter.

This study was aimed at examining whether methylation level of the *ABCA1* promoter influence the occurrence of pCAD and investigateing the relationship between *ABCA1* promoter methylation levels with lipid levels, inflammatory factors, and NETs in the Chinese pCAD patients, which will contribute us to explore the molecular pathogenesis of pCAD.

## Methods

### Study population

Consecutive patients with pCAD were recruited from the Department of Cardiology in the Characteristic Medical Center of the Chinese People's Armed Police Force from June to December 2019. PCAD was defined as a history of myocardial infarction, angioplasty, revascularisation surgery, or more than 50% stenosis in at least one coronary artery determined by coronary artery angiography and diagnosed before age 55 in men and 65 in women. The exclusion criteria included patients with cardiogenic shock, severe heart failure, severe liver and kidney dysfunction, a history of malignant tumour, and those undergoing treatment with lipid-lowering drugs or other drugs that affect blood lipid levels in the past 3 months. Finally, 90 pCAD patients were enrolled (52 with acute ST elevation myocardial infarction, 25 with acute coronary syndromes, and 13 with stable angina), and 25 patients were screened. Meanwhile, 112 sex- and age-matched asymptomatic individuals with no family history of CAD were recruited from the health examination department of our centre. Upon examination by coronary artery computed tomography or coronary angiography, we excluded 22 participants who at risk of progression to pCAD due to the coronary lesions < 50%. Finally, 90 individuals with normal coronary arteries were included in the control group.

The study was conducted in accordance with the Declaration of Helsinki and approved by the Ethics Committee of the Characteristic Medical Center of Chinese People's Armed Police Force (Clinical 2019–0001).

### Date collection and biochemical parameters

All participants underwent general health examination to measure height, weight, waist circumference, body mass index (BMI), and detailed information on medical (diabetes and hypertension) and treatment history and smoking habits were collected. Participants regularly smoking tobacco products at least once a day or those that have smoked 30 days prior to admission were considered as current smokers. Hypertension was diagnosed as systolic blood pressure ≥ 140 mmHg and/or diastolic ≥ 90 mmHg and/or undergoing antihypertensive treatment. Diabetes mellitus was considered when participants are taking glucose-lowering drugs due a previous diagnosis of diabetes according to the American Diabetes Association criteria [22]. Furthermore, hyperlipidaemia was defined as low-density lipoprotein cholesterol (LDL-C) > 130 mg/dl, total cholesterol (TC) > 200 mg/dl, triglyceride (TG) > 150 mg/dl, or high-density lipoprotein cholesterol (HDL-C) < 40 mg/dl according to the American College of Cardiology (ACC) and the American Heart Association (AHA) guidelines [23].

Venous blood samples were collected after an overnight fast of at least 8 h and assessed at the laboratory of our centre. Plasma glucose, TC, TG, HDL-C, and LDL-C were enzymatically assayed using a Siemens automatic biochemical analyser (Siemens ADVIA 2400 Chemistry System, Germany).

### Analysis of *ABCA1* promoter methylation by pyrosequencing

Pyrosequencing was conducted as previously described to detect the methylation status of the CpG islands in the promoter region of *ABCA1* [14, 24]. Primers for methylation analysis and PCR were designed (Table 1) using the PyroMark Assay Design software v2.0.1.15 (QIAGEN, CA, USA, # 9019077). Bisulphite conversion of genomic DNA was performed using an EpiTect Plus DNA Bisulfite Kit (QIAGEN, CA, USA, # 59104). Polymerase chain reaction (PCR) amplification was conducted on an ABI 9700 PCR System (Applied Biosystems, Foster, CA, USA) with a total reaction volume of 50 µL containing 2 µL bisulphite-converted DNA, 1 µL PCR primers, 2 µL dNTP mix, 10 µL 5*PCR GC buffer, and 0.2 µL Taq DNA polymerase (KAPA Biosystems, Boston, USA). The following PCR protocol was performed: 95 °C for 3 min, 45 cycles of (94 °C for 30 s, 50 °C for 30 s, and 72 °C for 1 min), followed by 7 min hold at 72 °C. The purified products, along with the substrate mixture, enzyme, and the four dNTPs (QIAGEN, CA, USA), were collected and placed in PyroMark Q48 Autoprep (QIAGEN, CA, USA,# 974022), for the pyrosequencing. Pyro Q-CPGTM software 2.0 (QIAGEN, CA, USA, # 9019077) was used to automatically analyse the methylation status of each site.

**Table 1:** PCR and pyrosequencing primer design

Amplification locus	Primer sequence	Length (bp)
*ABCA1*-A (8 CpGs)	F: 5′-GGGTGGAGGGTATAGTAGGT-3′	161
	R: 5′-AACAAATTCCACTAATACCCTTAACT-3′	
	Seq: 5′-AACAAATTCCACTAATACCCTTAACT-3′	

*ABCA1*, ATP-binding cassette transporter A1

### Measurement of serum ABCA1 concentration

The ABCA1 concentration in the blood samples was determined using an enzyme-linked immunosorbent assay (ELISA) kit (A268 SC, ELIXIR Canada Medicine Company, HCB, Canada) following the manufacturer's instructions. The average optical density of each sample was determined using an xMark™ Microplate Spectrophotometer (Bio-Rad, Hercules, CA, USA); the four-parameter equation was fitted; and the serum ABCA1 concentration in each sample was calculated based on the equation of the standard curve.

### Analysis of inflammatory factors

CRP was determined by turbidimetric immunoassay performed on an automated-analyser (Roche/Hitachi 902, TKY, Japan) using a CRP diagnostic kit (Mlbio, Shanghai, China). The level of IL-1β was detected by ELISA assay using a commercially available kit (Mlbio, Shanghai, China).

### NETs marker-cfDNA component testing

NETs comprise circulating free DNA (cfDNA), which can activate and amplify the immune response to an immunogen in a proinflammatory manner. Therefore, cfDNA can be used as a surrogate marker to assess the extent of inflammation in the body. The cfDNA/NETs component was measured as previously described [25]. Quant-iT PicoGreen dsDNA fluorescent dye (Invitrogen, Life Technologies, Carlsbad, CA, USA) was used to detect free DNA in the peripheral venous plasma. Briefly, the experimental solution was diluted 50× with TE buffer, mixed with 100 μL of an equal volume of Picogreen, and added into 96-well plates. The final concentrations of the standard products were 1, 0.1, 0.01, 0.001 and 0 μg/mL. The intensity of the signal was detected using a fluorescence spectrometer (LS55, Perkin Elmer, MD, USA) at an excitation wavelength of 480 nm and an emission wavelength of 520 nm.

### Statistical methods

SPSS19.0 software was utilised for statistical analysis. Normality was assessed using the Kolmogorov–Smirnov test. Normally distributed data are expressed as the mean ± SD and analysed using an independent *t*-test, whereas non-normally distributed data are expressed as the median and interquartile spacing and analysed using the Mann–Whitney test. Categorical variables were compared using Chi-square test. Correlation was evaluated using the Pearson's and nonparametric Spearman's rank correlation test. The independent association of each risk factor with pCAD was analysed using binary logistic regression. Factors affecting the methylation of *ABCA1* promoter were analysed using multiple regression analysis. *P* values < 0.05 were considered statistically significant.

## Results

### Characteristics and biochemical data of the pCAD and control groups

There were no significant differences in age, sex, smoking rate, systolic and diastolic blood pressure, TC, TG, LDL-C, fasting blood glucose, homocysteine (Hcy), and the incidence of hypertension between the two groups (*P* > 0.05). The levels of HDL-C were significantly lower (*t* = 9.477, *P* < 0.001) in the pCAD group than those in the control group, whereas BMI, waist circumference and HbA1c levels were significantly higher in the pCAD group (Table 2). PCAD patients were also more likely to have diabetes than the control group, whereas there was no significant difference in the duration of diabetes (control: 4.2 (2.1–6.5) years *vs.* pCAD: 5.1 (2.3–7.4) years) and the rates of insulin (control: 22.2% *vs.* pCAD: 24.4%) and hypoglycaemic drug (control: 26.7% *vs.* pCAD: 33.3%) used between the two groups (*P* > 0.05).

**Table 2:** Demographic and biochemical data of pCAD and control groups

Characteristics	Control group (n = 90)	PCAD group (n = 90)	*t/χ2/Z*	*P* value
Men (%)	54 (60.0)	61 (67.8)	1.180	0.277
Age (years)	53.43 ± 3.23	52.13 ± 5.35	1.975	0.051
BMI (kg/m^2^)	23.98 ± 1.34	24.98 ± 1.25	5.163	0.0005
Waist circumference (cm)	79.6 ± 3.50	80.96 ± 3.73	2.516	0.013
Cigarette smoking (%)	31 (34.4)	32 (35.6)	0.024	0.876
SBP (mmHg)	130.0 (125.1–145.5)	135.5 (130.0–150.0)	1.153	0.249
DBP (mmHg)	80.0 (75.0–88.5)	82.5 (75.0–90.0)	1.010	0.313
HDL-C (mmol/L)	1.19 ± 0.27	0.86 ± 0.18	9.477	< 0.001
LDL-C (mmol/L)	2.87 ± 0.82	3.00 ± 0.98	1.320	0.189
TC (mmol/L)	4.23 ± 0.84	4.41 ± 1.09	1.239	0.217
TG (mmol/L)	1.88 ± 1.67	2.11 ± 1.40	0.998	0.320
Hcy (μmol/L)	13.84 ± 3.15	14.58 ± 4.17	1.337	0.183
Fasting glucose (mmol/L)	5.55 (5.08–6.68)	5.90 (4.90–7.88)	0.780	0.435
HbA1c (%)	5.70 (5.20–6.33)	6.25 (5.40–7.13)	2.770	0.006
Diabetes mellitus (%)	26 (28.9)	41 (45.6)	5.349	0.021
Hypertension (%)	40 (44.4)	50 (55.6)	2.222	0.136
CRP (mg/L)	4.47 ± 3.06	7.44 ± 3.63	5.925	< 0.001
cfDNA/NETs (ng/mL)	9.93 ± 10.73	24.40 ± 13.18	8.064	< 0.001
IL-1β (pg/mL)	47.66 ± 14.77	57.74 ± 12.55	4.933	< 0.001

Variables are presented as means ± standard deviations, median (interquartile spacing), or percentages. BMI, body mass index; SBP, systolic blood pressure; DBP, diastolic blood pressure; HDL-C, high-density lipoprotein cholesterol; LDL-C, low-density lipoprotein cholesterol; TC, total cholesterol; TG, triglycerides; Hcy, homocysteine; HbA1c, glycated haemoglobin; CRP, C-reactive protein; cfDNA/NETs, circulating free DNA/Neutrophil extracellular traps; IL-1β, interleukin-1β

### Association between the *ABCA1* promoter methylation levels, serum ABCA1 concentration, and pCAD

**Fig. 1:**
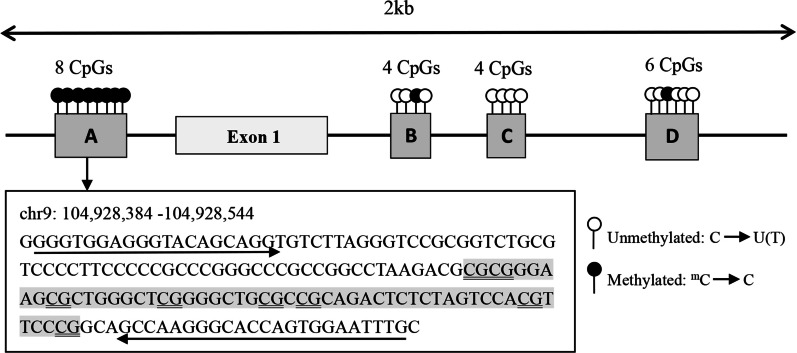
*ABCA1* CpG islands proximal promoter region. Arrows indicate PCR primer sequences. The epigenotyped region is shown in grey shading and the analysed eight CpG dinucleotides have been double-underlined

Similar to the results of Guay et al. [14, 24], only eight CpGs upstream the first exon of the *ABCA1*-A locus were selected for detection in Fig. 1, however, the sites from *ABCA1*-B to *ABCA1*-D were not evaluated as they have very low methylation levels. Our results showed that the DNA methylation levels of eight CpGs and the mean methylation levels of the promoter region of *ABCA1-A* were significantly higher in the pCAD group (*P* < 0.001), whereas the serum ABCA1 concentration was lower in the pCAD group than that in the control group (Table 3); and there was no correlation between the methylation level of the *ABCA1* promoter and the serum ABCA1 concentration (*r* = 0.146, *P* = 0.05).

**Table 3:** DNA methylation differences in eight CpG sites within the *ABCA1*-A locus in the two groups

Item	Control group (n = 90)	PCAD group (n = 90)	*t*	*P* value
CpG1 (%)	35.40 ± 3.87	43.54 ± 5.78	11.092	< 0.001
CpG2 (%)	25.61 ± 2.93	32.21 ± 5.99	9.396	< 0.001
CpG3 (%)	58.84 ± 6.53	71.75 ± 5.69	14.137	< 0.001
CpG4 (%)	39.76 ± 4.73	49.65 ± 8.38	9.751	< 0.001
CpG5 (%)	37.35 ± 8.36	44.03 ± 8.56	5.296	< 0.001
CpG6 (%)	29.93 ± 4.29	37.64 ± 5.68	10.282	< 0.001
CpG7 (%)	34.95 ± 4.20	42.43 ± 5.80	9.912	< 0.001
CpG8 (%)	27.11 ± 2.71	32.83 ± 4.88	9.714	< 0.001
Mean *ABCA1* promoter methylation (%)	36.05 ± 2.99	44.24 ± 3.66	16.429	< 0.001
ABCA1 concentration (ng/mL)	3.35 (3.02–3.95)	3.15 (2.73–3.74)	2.458	0.014

Variables are presented as means ± standard deviations and median (interquartile spacing)

To identify the independent association of each variable with the risk of pCAD, binary logistic regression analysis was performed. Upon considering the study group (pCAD *vs.* control) as the dependent variable and age, sex, BMI, waist circumference, TG, TC, HDL-C, LDL-C, HbA1c, diabetes, hypertension, and *ABCA1* promoter methylation status as the covariates, we identified the methylation level of the *ABCA1* promoter (odds ratio (OR) = 2.878, 95% confidence interval (CI) 1.802–4.594; *P* < 0.001), HDL-C (OR = 0.015, 95% CI 0.004–0.032; *P* < 0.001), BMI (OR = 1.892, 95% CI 1.374–2.604; *P* = 0.017) and HbA1c (OR = 3.162, 95% CI 1.148–8.709; *P* = 0.026) as independent risk factors for pCAD development (Table 4).

**Table 4:** Logistic regression analysis with factors potentially related to pCAD

Covariate	B	Standard Error	Wald	Sig	Exp (B)	95% CI for Exp (B)
Mean *ABCA1* promoter methylation level	1.057	0.239	19.601	< 0.001	2.878	1.802 ~ 4.594
HDL-C	− 9.565	2.711	12.452	< 0.001	0.015	0.004 ~ 0.032
BMI	1.180	0.495	5.692	0.017	1.892	1.374 ~ 2.604
HbA1c	1.151	0.517	4.960	0.026	3.162	1.148 ~ 8.709

The model was adjusted for the following co-variables: age, sex, Waist circumference, cholesterol, triglycerides, LDL-C, diabetes and hypertension, P > 0.05. HDL-C, high-density lipoprotein cholesterol; BMI, body mass index; HbA1c, glycated haemoglobin; Sig, significance; CI, confidence interval

### Correlation between HDL-C and *ABCA1* promoter methylation levels

**Fig. 2:**
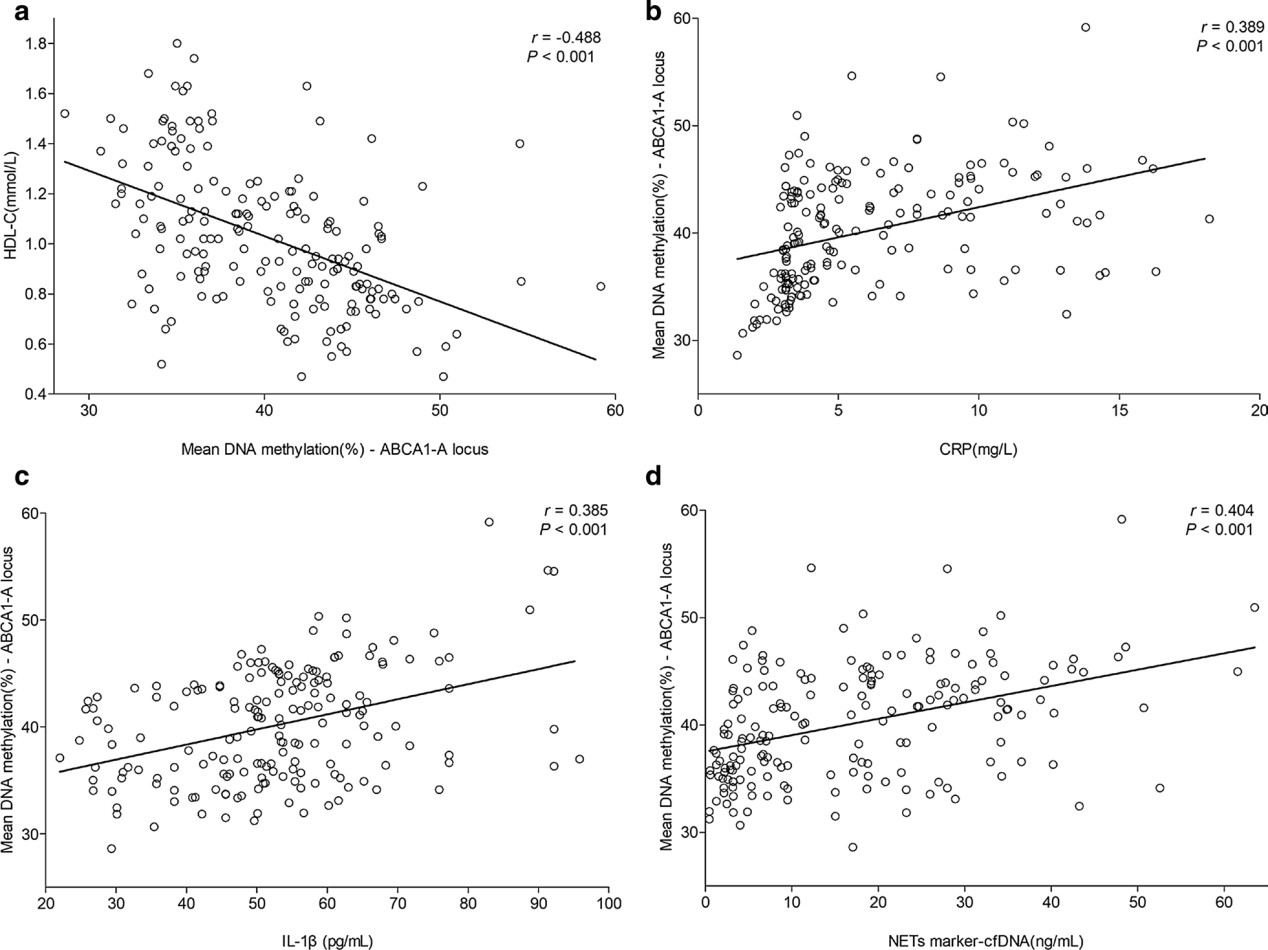
Serum levels of HDL-C and inflamatory markers according to *ABCA1* promoter methylation levels. Correlations between mean methylation levels (*ABCA1*-A locus) and serum levels of **a** HDL-C, **b** CRP, **c** IL-1β, and **d** NETs marker-cfDNA

*ABCA1* promoter methylation levels were negatively correlated with serum HDL-C levels (*r* =  − 0.488, *P* < 0.001) (Fig. 2a); but were not correlated with serum levels of LDL-C (*r* = 0.059, *P* = 0.430), TC (*r* = 0.099, *P* = 0.187), or TG (*r* = 0.047, *P* = 0.531).

### Correlation between *ABCA1* promoter methylation levels and plasma CRP, IL-1β, and cfDNA/NETs levels

As shown in Table 1, serum CRP, cfDNA/NETs, and IL-1β levels in the pCAD group were significantly higher than those in the control group and positively correlated with the average methylation level in promoter region of *ABCA1-A* (*r* = 0.389, *P* < 0.001; *r* = 0.404, *P* < 0.001; and *r* = 0.385, *P* < 0.001, respectively) (Fig. 2b–d). The covariates in Multiple regression model was established according to the univariate regression analysis and clinical significance, and the result showed that after adjusting for age, sex, smoking, and diabetes, serum CRP, cfDNA/NETs, IL-1β, and homocysteine levels remained as independent factors affecting the methylation level of the *ABCA1* promoter (Table 5).

**Table 5:** Multiple regression analysis with factors potentially related to the methylation levels of the *ABCA1* promoter

Constant	Unstandardised coefficients		Standardised coefficients	t	Sig	95% confidence interval for B
	B	Std. Error	Beta			
HCY	0.187	0.090	0.131	2.085	0.039	0.010 ~ 0.364
CRP	0.303	0.098	0.210	3.103	0.002	0.110 ~ 0.495
cfDNA/NETs	0.087	0.025	0.229	3.444	0.001	0.037 ~ 0.136
IL-1β	0.079	0.024	0.218	3.257	0.001	0.031 ~ 0.127

The model was adjusted for co-variables such as age, sex, smoking history and diabetes, *P* < 0.05*.* HCY, homocysteine; CRP, C-reactive protein; cfDNA/NETs, circulating free DNA/Neutrophil extracellular traps; IL-1β, interleukin-1β; Sig, significance

### Association between average methylation level of *ABCA1* promoter, age, and sex

**Fig. 3:**
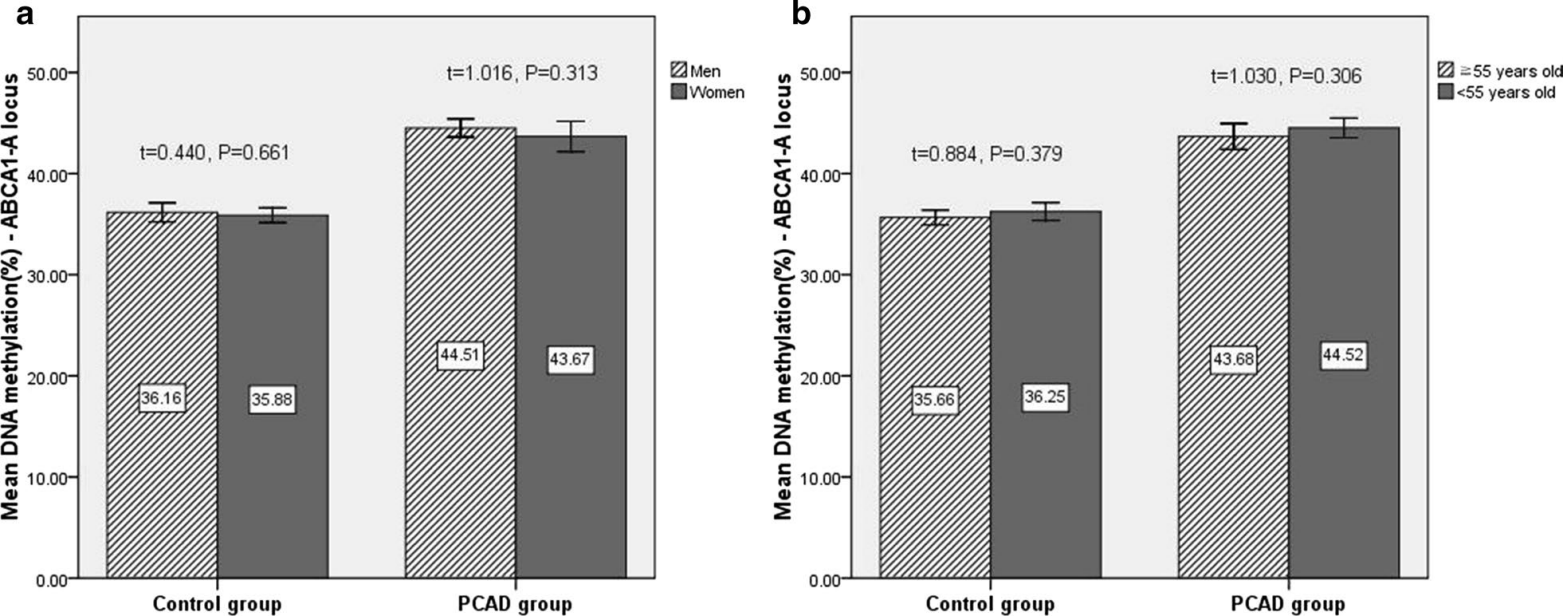
Comparison of average levels of *ABCA1* promoter methylation according to gender (**a**) and age (**b**)

We further assessed whether age and sex are associated with the mean methylation level of the *ABCA1* promoter. As evident in Fig. 3a, b, there were no significant differences in the average methylation levels between the male and female groups and between the ≥ 55-year-old and < 55-year-old subjects (*P* > 0.05).

## Discussion

As the main pathophysiological basis of CAD, AS manifests as chronic inflammation and the development of AS involves abnormal DNA methylation [26]. A previous study has demonstrated that in FH patients, peripheral blood leukocyte *ABCA1* promoter methylation levels and plasma HDL-C levels are negatively correlated and that FH patients with CAD exhibit higher *ABCA1* promoter methylation levels than those without [14]. Moreover, the degree of *ABCA1* promoter methylation was significantly correlated with the age and the incidence of CAD [27]. However, another study by Ghaznavi et al. [15] in an Indian population has indicated that there is no correlation between *ABCA1* promoter methylation levels and the severity of CHD and that the effect of *ABCA1* promoter methylation levels on CHD is independent from its effect on plasma lipid levels.

This study established the following: (1) Compared with the control group, the average methylation level of the *ABCA1* promoter in pCAD patients were significantly higher, whereas the level of HDL-C and serum ABCA1 concentration were lower; (2) The methylation level of the *ABCA1* promoter was an independent risk factor for pCAD, along with traditional risk factors, such as low HDL-C, high BMI and HbA1c; and (3) *ABCA1* promoter methylation levels were negatively associated with the plasma HDL-C levels. These results imply that, on the one hand, *ABCA1* promoter methylation regulates its mRNA expression by inhibiting the transcription of the *ABCA1* promoter, resulting in decreased levels of ABCA1, which leads to the inactivation of RCT and the acceleration of lipid deposition. Moreover, many risk factors affect pCAD, such as smoking, dyslipidaemia, diabetes, hypertension, and family history of CAD [28]. We also found that the methylation level of the *ABCA1* promoter increases the risk of pCAD independently of the lipid level, and the genetic predisposition may play a key role. Therefore, the methylation level of the *ABCA1*-A promoter may be used as a potential biomarker to predict the occurrence risk of pCAD in clinical practice and to assist in the early diagnosis, treatment, and monitoring of pCAD in a non-invasive manner.

DNA methylation levels are influenced by heredity, age, smoking, nutritional status, and environment [29–32] and closely related to inflammatory responses, which have been the focus of many studies in recent years [33]. DNA methylation plays a regulatory role during inflammatory responses, mainly through the regulation of inflammatory cytokines [34] and inflammatory signalling pathways [35]. In addition, some inflammatory factors can promote the expression of DNMTs and increase their activity, thereby causing abnormal methylation of DNA [36]. Recent evidence suggests that inflammation can interact with RCT processes and that ABCA1 is a key regulatory factor in this interaction. The deletion of ABCA1 in mice resulted in increased cholesterol in the lipid rafts on the membrane of macrophages, and the activation of inflammatory signalling pathways through the Toll-like receptor 4 (TLR4) led to an increase in the levels of inflammatory factors [37]. Westerterp et al. [38] found that in the early stages of AS, ABCA1/G1 deficiency in the progenitor cells of the bone marrow may result in the activation of the inflammasome, neutrophil infiltration into atherosclerotic plaques, and the increased release of NETs, ultimately promoting AS. Therefore, as stimulators of macrophage inflammasomes, NETs are closely related to hypercholesterolemia induced by decreased ABCA1 expression and cholesterol effusion.

Our study found that *ABCA1* promoter methylation level was positively associated with serum inflammatory factors (CRP, IL-1β) and cfDNA/NETs; meanwhile, the patients of pCAD group had higher serum levels of inflammatory cytokines and cfDNA/NETs than the control group, suggesting that inflammatory responses contribute to the promotion of pCAD. The reason may be due to the changes in the bodily environment of patients with pCAD that can cause epigenetic modifications, resulting in increased methylation of the associated genes and increased activity of DNA methyltransferases [39], thus disrupting gene expression, decreasing ABCA1 levels, and weakening the inhibitory effects of inflammatory factors. Meanwhile, increased serum inflammatory factor levels in patients with pCAD could inhibit the expression and function of ABCA1, and reduce the efficiency of RCT, thereby reducing HDL levels. Therefore, we hypothesise that the increased methylation of *ABCA1* promoter in pCAD patients might affect the expression of ABCA1, which interacts with inflammatory factors to stimulate the development of early coronary AS.

In addition, increasing evidence shows that epigenetic processes are not static and that DNA methylation is a reversible process that can be regulated. Lou et al. [40] has revealed that the use of resveratrol can change the levels of DNA methylation and reduce inflammatory factors at specific CpG sites, indicating the therapeutic role of resveratrol in diabetic vascular complications. Our study showed that the levels of serum CRP, IL-1β and cfDNA/NETs are independent factors affecting *ABCA1* promoter methylation, which may verify clinically that inflammatory mediators can further promote the development of pCAD via regulating DNA methylation. Therefore, we speculate that intervention with pharmaceuticals exerting anti-inflammatory activities during the early course of pCAD may regulate the methylation status of *ABCA*1, and can be applied to the development of therapeutic strategies for pCAD. However, more studies are needed to confirm our hypothesis.

Furthermore, unlike a previous study [27], our study found no significant relationship between age and *ABCA1* promoter methylation level. We attribute this to our study population that was limited to patients with pCAD and the relatively narrow age span. Another study has showed that the methylation levels was more significantly different in elderly patients with coronary heart disease > 65 years old than those younger [15], suggesting that age might not be the main factor involved in the increased methylation status of pCAD patients.

### Study limitations

First, due to the specificity of the age of onset of pCAD and the high cost of pyrosequencing analysis, our sample size was relatively small, and our participants were recruited from a single centre within a stable population. Second, we did not find any correlation between the serum ABCA1 concentration and the methylation level of *ABCA1* promoter due to the low ABCA1 concentration in the peripheral blood. The expression of *ABCA1* mRNA need to be further investigated. Third, as the definition of pCAD is not uniformly established, and consider the actual situation of our centre and various literature studies [4–7], we used the common age cut-off value (before age 55 in men and 65 in women) in China, especially for women, due to the large age range, our findings may be more applicable to the relatively young patients with CAD.

### Future directions

The incidence of pCAD has increased significantly along with improved living standards; however, the pathogenesis of pCAD is not well understood. In the future, a larger sample size is required for the assessment of the potential predictors of severity of pCAD and comparison with mature CAD. Meanwhile, further basic and clinical studies are warranted to confirm the role of inflammation on methylation.

## Conclusions

This study revealed the association between higher methylation levels at the *ABCA1* promoter and lower HDL-C plasma level and identified high *ABCA1* promoter methylation as an independent risk factor for pCAD development. Additionally, our findings suggest that inflammatory factors and NETs are involved in the progression of pCAD by affecting *ABCA1* promoter methylation levels. Therefore, the regulation of DNA methylation may serve as a novel therapeutic strategy for pCAD.

## Data Availability

The datasets generated and analysed during the current study are not publicly available due to fact that our project includes a series of studies on methylation that need to be continued, and some of the participants were recruited from the military, whose data were required to be confidential, but are available from the corresponding author on reasonable request.

## Abbreviations

ABCA1: ATP-binding cassette transporter A1
RCT: Reverse cholesterol transport
pCAD: Premature coronary artery disease
CRP: C-reactive protein
cfDNA: Circulating free DNA
CAD: Coronary atherosclerotic heart disease
HDL: High-density lipoprotein
LDL: Low-density lipoprotein
BMI: Body mass index
AS: Atherosclerosis
DNMT: DNA methyltransferase
FH: Familial hypercholesterolemia
NETs: Neutrophil extracellular traps
IL-1β: Interleukin-1β
